# Age-related decline in positive emotional reactivity and emotion regulation in a population-derived cohort

**DOI:** 10.1093/scan/nsz036

**Published:** 2019-05-23

**Authors:** Susanne Schweizer, Jason Stretton, Janna Van Belle, Darren Price, Andrew J Calder, Cam-CAN, Tim Dalgleish

**Affiliations:** 1 Medical Research Council Cognition and Brain Sciences Unit, Cambridge, CB2 7EF, UK; 2 Institute of Cognitive Neuroscience, University College London, London, WC1N 3AZ, UK; 3 Cambridgeshire and Peterborough NHS Foundation Trust, Cambridge, CB21 5EF, UK

**Keywords:** positivity, emotion regulation, ageing, socioemotional selectivity theory, middle frontal gyrus

## Abstract

Human older age ushers in functional decline across the majority of cognitive domains. A notable exception seems to be affective processing, with older people reporting higher levels of emotional well-being. Here we evaluated age-related changes in emotional reactivity and regulation in a representative subsample (*N* = 104; age range: 23–88 years) of the population-derived Cambridge Centre for Ageing and Neuroscience cohort. Performance on a film-based emotion reactivity and regulation task in the magnetic resonance imaging scanner showed an age-related decline in positive reactivity, alongside a similar decline in the capacity to down-regulate negative affect. Decreased positivity with age was associated with reduced activation in the middle frontal gyrus. These findings, from the largest neuroimaging investigation to-date, provide no support for age-related increases in positive emotional reactivity.

Increased positivity in older age seems to be a paradoxical finding considering the ubiquitous cognitive and physical decline across other domains of functioning (Hedden and Gabrieli, 2004; Hartshorne and Germine, 2015), yet it has received much previous empirical support from psychological and neuroscientific research (Carstensen, 2006; Mather, 2016; Carstensen and DeLiema, 2018). The most prominent theoretical account of age-related positivity is Carstensen’s Socioemotional Selectivity Theory (SST; 1995, 2006). SST proposes a motivational shift in the elderly precipitated by an age-related enhanced awareness and acceptance of the finiteness of life. As a consequence, motivation shifts away from goals of knowledge acquisition and social networking that prepare younger people for uncertain futures, towards more immediate goals that emphasise emotional meaning and savouring of everyday experiences. SST proposes that this motivational shift impacts the choices people make, with older individuals being less willing to engage in situations that immediately elicit unwanted negative affect, even though they may potentially confer benefits for longer-term achievement-related or status-related goals (Carstensen *et al.,* 2003; Blanchard-Fields, 2007; Charles, 2010; Rovenpor *et al.,* 2013).

Alongside this behavioural selectivity towards pleasant experiences in older age, there is robust evidence for age-related shifts in *cognitive* selectivity. In predominant laboratory studies, older adults have been shown to preferentially process positive over negative material across a range of cognitive domains including attention, working memory, autobiographical memory and decision-making (Mather and Carstensen, 2005; Carstensen, 2006; Reed *et al.,* 2014; Samanez-Larkin and Knutson, 2015; Mather, 2016). This age-related cognitive selectivity—termed the *positivity effect* (Carstensen and DeLiema, 2018)—is associated with elevated activation in prefrontal cortical (PFC) networks implicated in executive control (St Jacques *et al.,* 2009; Nashiro *et al.,* 2012; Mather, 2016).

The positivity effect has been widely replicated (Reed *et al.,* 2014). However, typically these studies compare discrete groups of older and younger participants rather than sampling across the lifespan (Reed *et al.,* 2014). Moreover, while it is implied that the positivity effect reflects increased positive emotional reactivity in response to positive stimuli, this is less well understood. Literature examining neural responses to positive stimuli more broadly has reliably implicated the ventral striatum and ventromedial PFC (Bartra *et al.,* 2013), in contrast to regions including the dorsal striatum, dorsomedial PFC and insula, which have been implicated in arousal and salience detection of positive and negative items. The primary aim of the current study was therefore to assess age-related changes in positive reactivity and its neural substrates using a population-derived sample drawn from across the lifespan.

We administered a film-based emotion reactivity and regulation task (Figure 1) in the magnetic resonance imaging (MRI) scanner to a subsample (*n* = 104, age range: 23–88 years) of a neuropsychiatrically healthy population-representative Cam-CAN (Shafto *et al.,* 2014) cohort. If older age is characterised by increased positivity, we would predict age-related increases in both reported positive affect and the engagement of brain networks in the PFC associated with cognitive control, when participants simply watch positive or negative (relative to neutral) film clips, as older participants should selectively process positive components of these complex, dynamic stimuli as a function of tonic emotional up-regulation processes.^.^

**Fig. 1:**
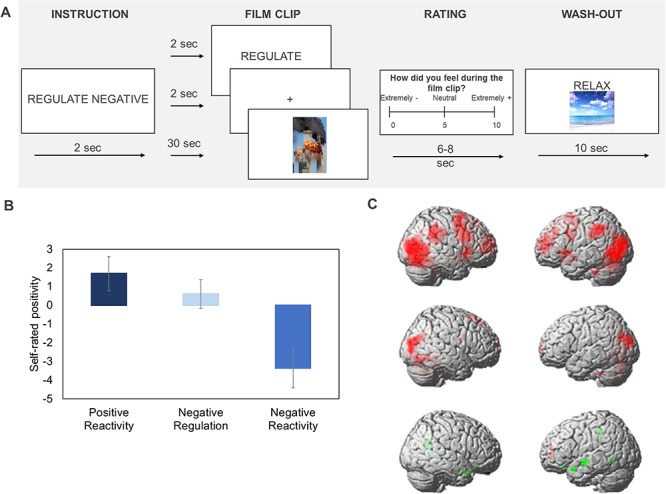
(**A**) A sample trial of the emotion regulation and reactivity task where participants viewed a set of negative, neutral or positive 30 second film clips. In the ‘watch’ condition, participants were asked to simply watch the films and allow their emotions to arise naturally. In the ‘regulate’ condition (example in A), participants were asked to reduce their emotions to a negative film by changing—reappraising—the way they thought about the film’s content. After each film clip, participants rated how they felt during the film on a scale from ‘0 = Extremely negative’ to ‘10 = Extremely positive’. After each positive or negative film clip, participants watched a brief washout clip to return their mood to normal. The task provides measures of: (i) Positive Reactivity—how positively participants experienced the positive (relative to neutral) film clips; (ii) Negative Reactivity—how negatively participants experienced the negative film (relative to neutral) clips and; and (iii) Negative Regulation—by how much participants were able to reduce their negative emotions when instructed to do so in the regulate relative to watch condition. Positive and Negative Reactivity were computed by subtracting the emotion reported in the neutral film, as a reactivity baseline, from emotions reported in the positive and negative watch conditions, respectively. Negative Regulation was computed by subtracting the emotion rating from the negative watch condition from the rating during the negative regulate condition (Schweizer *et al.,* 2013). Similar contrasts were applied to the fMRI data. (**B**) As a manipulation check, B shows that, collapsed across age, participants responded to the emotional films as expected with increased and reduced positivity, for positive and negative films, respectively, and successful down-regulation of negative emotion in the regulate condition (main effect of viewing condition *F* (2, 102) = 527.81, *P* < 0.001, η_p_^2^ = 0.91), with emotion ratings being highest for Positive Reactivity (*M* = 1.70, *SD* = 0.92), intermediate for the Negative Regulate condition (*M* = 0.60, *SD* = 0.77) and lowest in the Negative Reactivity condition (*M* = −3.35, *SD* = 1.04). See Supplementary Materials for ratings in the individual conditions. (**C**) In line with previous literature on the neural substrates of emotional reactivity and regulation (Lindquist *et al.,* 2012; Buhle *et al.,* 2014), Positive Reactivity was associated with greater activation (red, top row) in bilateral middle frontal gyrus, left anterior insula cortex, occipital cortex, thalamus and medial prefrontal cortex (Table S1). Negative Reactivity was associated with greater activation (red, middle row) in the occipital cortex, precuneus, left caudate and the right medial, middle and superior frontal cortices (Table S1). Finally, Negative Regulate was associated with greater activation (red, bottom row) in the left middle frontal gyrus (Table S1) and reduced (contrast negative regulate < negative watch) activation (green, bottom row) in various regions including the subgenual prefrontal cortex, right temporal pole extending into the amygdala, left superior temporal gyrus and precuneus/posterior cingulate cortex (Table S1), indicating that these regions were down-regulated in the Negative Regulate condition.

The second aim of the current study was to examine age-related differences in the ability to effortfully down-regulate negative affect when explicitly instructed to do so. Research investigating the relationship of age to the effortful regulation of emotion under laboratory conditions has yielded mixed results, including age-related improvements (Phillips *et al.,* 2008; Lohani and Isaacowitz, 2014), no age effects (Martins *et al.,* 2014; Opitz *et al.,* 2014; Allard and Kensinger, 2018) and reduced regulatory abilities (Opitz *et al.,* 2012; Tucker *et al.,* 2012), with concomitantly greater PFC activation in younger adults (Winecoff *et al.,* 2011; Opitz *et al.,* 2012; Allard and Kensinger, 2014; Martins *et al.,* 2014).

We examined age-related effects on self-reported affect and activation across brain networks implicated in effortful emotion regulation (Buhle *et al.,* 2014). We predicted that the enhanced neurocognitive capacity for cognitive control associated with younger age would lead to age-related decline in the ability to effortfully regulate negative emotions, with concurrent reductions in activation in associated brain networks.

## Methods

### Participants

**Fig. 2:**
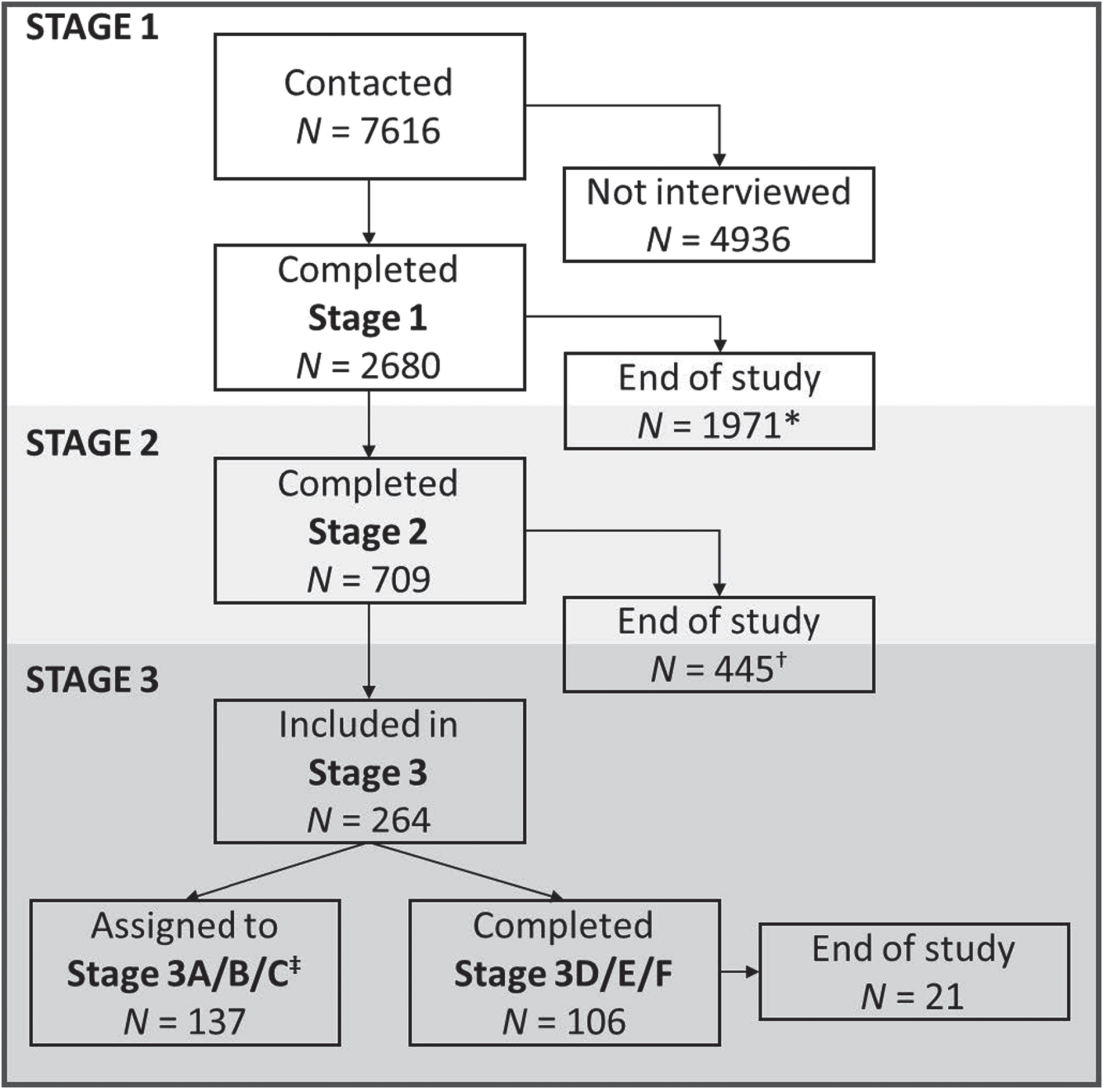
Participant inclusion stream for individuals who were contacted. ^*^N = 1528 individuals were excluded from Stage 2 by the computer algorithm ensuring random sampling across each decile; ^†^*N* = 3 individuals were excluded from Stage 3 by the computer algorithm ensuring random sampling across each decile; ^‡^Stage 3A/B/C is not discuss here further as it was not included in the current study.

Participants were a cohort-representative subsample (*n* = 106), targeted for in-depth neurocognitive phenotyping, derived from the healthy, population-derived Cam-CAN cohort (Shafto *et al.,* 2014). The Cam-CAN study was conducted in three stages (Figure 2). In Stage 1 7616 individuals were approached at their homes to complete a detailed interview and cognitive assessment (for details see below and Shafto *et al.,* 2014). In Stage 2, 100 participants from each decile 18–88 were invited to do further cognitive testing see (Shafto *et al.,* 2014), exclusion criteria at this stage were low Mini Mental State Examination (24 or lower), poor hearing (failing to hear 35 dB at 1000 Hz in either ear), poor vision (below 20/50 on Snellen test), poor English knowledge (non-native or non-bilingual English speakers), self-reported substance abuse and current serious health conditions (e.g. self-reported major psychiatric conditions, current chemo/radiotherapy or a history of stroke). In Stage 3 40 individuals per decile were invited to participate in functional neuroimaging tasks (Shafto *et al.,* 2014). Due to the high number of tasks, Stage 3 was split across three assessment sessions. In the first two sessions all participants completed the same tasks. In the third session half the participants received on subset (3C in Figure 2) of tasks and the other half of the participants completed the second subset (3F in Figure 2). Exclusion criteria for Stage 3 were as follows: safety- and health-related contraindications (e.g. disallowed implants, pacemakers, recent surgery or any previous brain surgery, current pregnancy, facial or very recent tattoos or a history of multiple seizures or fits) to MRI scanning as well as comfort-related issues (e.g. claustrophobia or self-reported inability to lay supine for an hour).

#### Sampling cohort

The sampling frame was the Primary Care Trust (PCT) population list of residents in the Cambridge City area. This constitutes a strong reflection of the general population as general practitioner (GP) registration is virtually universal in the UK’s health care system (Shafto *et al.,* 2014). From this list a potential sample of *N* = 20 895 was ascertained. A total of 13 279 of whom were not eligible due to incorrect data in the PCT (*N* = 3621, 27%), individuals falling outside the study inclusion (see above) criteria (*N* = 5010, 38%) and individuals with whom no contact was made after up to three visits (*N* = 4648).

Our ethical approval allowed Cam-CAN experimenters to make direct contact with the remaining 7616 individuals without prior individual consent. Figure 2 outlines the inclusion/exclusion flow for these individuals. Table 2 provides further details on reasons for exclusion for individuals who opted out/were unable to participate in the study for each study stage separately. Of the 127 individuals who were randomised to Stage 3D/E/F, 9 individuals completed only sessions D and E; one participant felt unwell as they entered the scanner; three participants refused to complete the task because of the emotional stimuli; two participants aborted the task during the scan because they found the films too distressing; four participants did not complete the scan because they ran out of time during the testing session; for one participant there was an equipment failure; and all information was missing for one participant.

**Table 1:** Reasons given for not participating in each study stage of the Cam-CAN study

***N* (%)**	**Did not complete stage1**	**Did not complete stage 2**	**Not included in stage 3**
*Active refusal (opt out)*	3008 (61)	130 (29)	146 (33)
No reason given	860 (28)	20 (15)	3 (2)
Too busy	1169 (39)	52 (40)	14 (10)
Not interested	835 (28)	1 (1)	2 (1)
Disagrees with science	84 (3)	—	—
Other	60 (2)	57 (44)	127 (87)
*Passive refusal*	164 (3)	—	—
Relative	102 (62)	—	—
Residential/care home	26 (16)	—	—
Neighbour	13 (8)	—	—
Not at appointment	23 (14)	—	—
*Illness*	1756 (36)	11 (3)	13 (3)
*Change in circumstances*	—	76 (17)	77 (17)
Died	—	4 (5)	2 (2)
Moved	—	9 (12)	33 (43)
MRI/MEG compatibility		40 (53)	29 (38)
Other	—	23 (30)	13 (17)
*Oversampled*	—	210 (47)	190 (43)
*Missing information*	8 (0)	16 (4)	16 (4)

Passive refusal = refusal by someone other than the participant; illness = too ill to participate as indicated by participant, proxy or GP; MRI/MEG compatibility = these participants were no longer compatible to be scanned in the MRI scanner or the magnet encephalograph; oversampled = individuals in excess of the 700 and 280 targets for stages 2 and 3, respectively.

#### Current sample

Two subjects were removed from the final analysis as defined outliers (> 3 *SD* above the mean for ratings across all emotion regulation task scales), leaving a final sample of 104 subjects (see Table 1). The study sample did not differ from the overall Cam-CAN sample (*N* = 2680) in terms of gender distribution (50% female *vs* 57%; *Χ^2^* = 2.99, *P* = 0.22), total years in education (*M* = 21.05, s.d. = 3.30 *vs M* = 19.86, s.d. = 5.39; *t* (2631) = 1.80, 95% [−0.11; 2.49], *P* = 0.072, *R^2^* = 0.00) or symptoms of depression and anxiety as measured by the Hospital Depression and Anxiety Scale (Zigmond and Snaith, 1983) (*M* = 9.51, s.d. = 5.40 *vs M* = 8.61, s.d. = 4.76; *t* (2597) = −1.68, 95% [−1.96; 0.15], *P* = 0.093, *R^2^* = 0.00). The study sample, however, was significantly younger than the overall Cam-CAN cohort, (*M* = 53.15, s.d. = 17.51 *vs M* = 60.84, s.d. = 20.99; *t* (2674) = −3.69, 95% [−1179; −3.60], *P* < 0.001, *R^2^* = 0.01), as the age and/or frailty of the older Cam-CAN participants meant they were disproportionately likely to meet study exclusion criteria. Nevertheless, the age range of the study sample was 23–88 years (see Table 2) and within that range the subsample remained representative of the wider sample.

**Table 2:** Participants’ (*N* = 104) demographic and cognitive characteristics

Decade	1	2	3	4	5	6	7
*n*	10	15	18	19	18	13	11
Age range; *M* (*sd*)	23–27; 25.8 (1.3)	28–38; 33.5 (3.4)	39–47; 43.1 (2.6)	48–57; 52.8 (3.1)	59–68; 64.1 (2.6)	68–77; 71.7 (2.8)	79–88; 81.8 (3.1)
Female *n* (%)	5 (50)	9 (60)	8 (44)	9 (47)	7 (39)	7 (54)	7 (63)
Education *n* (%)							
None			1 (5)	1 (5)		2 (15)	1 (9)
GCSE	1 (10)	2 (13)	2 (12)	1 (5)	2 (12)	3 (23)	1 (9)
A-level			1 (5)	4 (21)	1 (5)		1 (9)
University degree	9 (90)	13 (87)	14 (78)	13 (69)	15 (83)	8 (62)	8 (73)
Fluid intelligence *M* (*sd*)	38.9 (2.8)	38.8 (5.4)	34.4 (4.3)	32.8 (4.5)	32.7 (5.7)	26.9 (5.5)	28.1 (5.5)

None = no formal education; GCSE = General Certificate of Secondary Education (taken at age 16 years, after 9 years of formal education) and is equivalent to a US high-school diploma; A-level = General Certificate of Education Advanced Level is taken at age 18 years after 11 years of formal education and is equivalent to the International Baccalaureate; fluid intelligence = total score on the Cattell Culture Fair test of intelligence (Cattell, 1971).

## Materials

### Emotion reactivity and regulation

Emotion reactivity and regulation were assessed with a film-based paradigm (Figure 1) (Schweizer *et al.,* 2013, 2016). Participants viewed a series of forty 30‐second film clips that were either positive (e.g. babies laughing), neutral (e.g. weather report) or negative (e.g. documentary of the Rwandan genocide) in valence and consisted of a mixture of real-life and fictional footage (see Supplementary Materials for details). Participants received one of two different viewing instructions before each clip. Either ‘watch’, where participants were told to watch the film clips and allow themselves to feel any emotions that naturally arose without trying to deliberately distract themselves from the content of the film clip or effortfully regulate their emotions in any way. The second viewing instruction ‘regulate’ was only applicable to half of the negative film clips. Here, participants were explicitly asked to try to reduce (down-regulate) any negative affect in response to the film clip by using reappraisal techniques to change the way they thought about the contents of the film clip.

Before each film clip, participants received a prompt to indicate the valence and viewing instruction for the clip (e.g. ‘watch neutral’ or ‘regulate negative’). This was followed by the clip itself, after which participants rated their emotional response on a scale ranging from ‘very negative’ (0) to ‘very positive’ (10). Instruction, film clip and affective rating together comprised an experimental trial. Finally, after viewing a positive or negative film clip (but not following neutral film clips), participants saw a calming film clip (e.g. waves gently rolling back and forth on a beach with a meditative soundtrack) before the next trial. This was to allow their mood to return to normal. The film clips were presented in the MRI scanner in one continuous experimental sequence that contained 10 experimental trials from each of four conditions (watch neutral, watch positive, watch negative, regulate negative); the presentation order of the trials was pseudo-randomised, such that participants always started with a neutral trial and ended with a positive trial.

### Neuroimaging data acquisition and pre-processing

#### Data acquisition

Data were collected from a Siemens 3 T TIM TRIO scanner (Siemens, Erlangen, Germany). A high-resolution T1-weighted structural image was acquired using a Magnetization Prepared RApid Gradient Echo (MPRAGE) sequence with the following parameters: repetition time (TR) = 2250 ms; echo time (TE) = 2.99 ms; inversion time (TI) = 900 ms; flip angle = 9 degrees; field of view (FOV) = 256 mm × 240 mm × 192 mm; voxel size = 1 mm isotropic; GRAPPA acceleration factor = 2; acquisition time of 4 minutes and 32 seconds. T2^*^-weighted fMRI images were acquired while participants performed the task, using a Gradient-Echo Echo-Planar Imaging (EPI) sequence. An average total of 910 volumes were acquired, each containing 32 axial slices (acquired in descending order), slice thickness of 3.7 mm with an interslice gap of 20% (for whole brain coverage including cerebellum; TR = 1970 ms; TE = 30 ms; flip angle = 78 degrees; FOV = 192 mm × 192 mm; voxel-size = 3 mm × 3 mm × 4.44 mm) and an average acquisition time of 29 minutes.

#### Data pre-processing

Data pre-processing was carried out in SPM 8 (www.fil.ion.ucl.ac.uk/spm) implemented in an AA 4.0 pipeline (https://github.com/rhodricusack/automaticanalysis). The pre-processing steps included the following: (i) spatial realignment to correct for head movement and movement by distortion interactions; (ii) temporal realignment of all slices to the middle slice; (iii) co-registration of the EPI to the participant’s T1 anatomical scan; (iv) unified-segmentation-normalisation to normalise the T1 image to the MNI template, the parameters of which were then applied to the functional data; and (v) spatial smoothing with an full width at half maximum (FWHM) of 8 mm to meet the lattice assumption of random field theory and account for residual inter-subject structural variability.

### Statistical analyses

#### Behavioural analyses

To investigate emotional reactivity and regulation we computed a set of indices in line with the previous literature (Schweizer *et al.,* 2013, 2016). Positive Reactivity was computed by subtracting emotion ratings in the neutral watch condition, as a reactivity baseline, from the positive watch condition. Negative Reactivity was computed by subtracting the same neutral watch score from the negative watch score. Finally, Negative Regulation was computed by subtracting the emotion rating from the negative watch condition from the negative regulate condition. To ensure that the film clips elicited the anticipated emotional reactions and that participants had been able to down-regulate their affect when instructed to do so, emotion ratings were entered into in a repeated measures analysis of variance across positive and negative reactivity and negative regulation (Figure 1B).

To investigate the effect of age on emotional reactivity and regulation, these three indices were entered into a multivariate linear regression analysis, which was followed-up by individual regression analyses for each condition. We next explored whether any effects of age could be accounted for by the anticipated age-related decline in fluid intelligence, approximated by scores on the Cattell Culture Fair test (Cattell, 1971). Finally, to test for non-linear relationships between age and behaviour we included a polynomial quadratic expansion term (age-squared) in a hierarchical regression model for all measures. None of these was significant and we therefore only report significant linear associations.

#### Imaging data analyses

At the first level, each participant’s pre-processed data were entered into a GLM, with a separate regressor determined for each of the task conditions (watch neutral, watch positive, watch negative, regulate negative), the washout clips and the rating scales. We also included six estimated realignment parameters of movement between scans as covariates of no interest. The previously described contrasts of each condition relative to the watch neutral baseline: Positive Reactivity (watch positive minus watch neutral), Negative Reactivity (watch negative minus watch neutral), Negative Regulation (regulate negative minus watch negative and watch negative minus regulate negative, to explore regions associated with up- and down-regulation across conditions), positive watch minus negative watch and negative watch minus positive watch, were generated and taken to the second level for random-effects analysis. At the second level, we performed a one sample t-test of each reactivity and regulation index contrast. To investigate the effects of age on neural activity during emotion regulation and reactivity, we ran separate additional analysis including age as a covariate.

Given the theoretically derived *a priori* predictions regarding the effect of age on amygdala and PFC activation we initially ran regions of interest (ROIs) analyses. The PFC regions included were selected based on a meta-analysis of reappraisal as an ER strategy (Buhle *et al.,* 2014), which was the explicit emotion regulation strategy employed in the current study. These ROIs also include regions implicated in fMRI studies of the positivity effect (Nashiro *et al.,* 2012). We defined 10 mm spheres around the peak activations for each of the PFC clusters identified in the meta-analysis of reappraisal using MarsBar (Brett *et al.,* 2002). The ROIs included were as follows: right inferior frontal gyrus (peak coordinates: 51/15/48), bilateral middle frontal gyrus (left: −33/3/54; right: 60/24/3), right medial frontal gyrus (9/30/39) and bilateral amygdala (left: −18/−3/−15, right: 30/−3/−15). We used an adjusted level of *α* = 0.008 (Bonferroni corrected *α* = 0.05/6 = 0.0083) to examine our ROIs for significance, to account for multiple tests.

As emotion regulation is typically evaluated in response to negative material, it was proposed during the peer review process that we additionally run exploratory analyses of the effect of age on activation in reward-related regions in the bilateral ventral and dorsal striatum as well as the ventromedial PFC (Liu *et al.,* 2011). The anatomical ROIs were derived from the NeuroImaging Tools and Research Collaboratory’s (NITRC; https://www.nitrc.org) Wake Forest University PickAtlas.

To explore the effects of age we entered the extracted mean levels of BOLD activation in each ROI for each reactivity and regulation index in a series of linear regression analyses including age as a predictor. The functional analyses were supplemented with exploratory volumetric analyses. The relevant methods and results are reported in the Supplementary Materials. As with the behavioural analyses we ran a test of non-linear relationships of age including age as polynomial quadratic regressor (age-squared) in the model. These analyses were not significant and are not reported further.

Finally, we conducted whole-brain analyses for each regulation and reactivity contrast, thresholded at *P* < 0.05 FWE corrected, to explore significant activations in brain regions outside of our ROIs.

### Procedure

Following informed consent, participants practiced the emotional reactivity and regulation task before performing the task in the scanner. Participants completed other tasks as part of the Cam-CAN assessment (Shafto *et al.,* 2014). Participants were compensated £10 per hour plus £3 for travel. This study complied with the Helsinki Declaration, and was approved by the Cambridgeshire 2 Research Ethics Committee (reference: 10/H0308/50).

### Data and software availability

All Cam-CAN data is freely available on request through the access portal (https://camcan-archive.mrc-cbu.cam.ac.uk/dataaccess/). All analyses code is available on the University of Cambridge, Medical Research Council Cognition and Brain Sciences Unit data repository.

## Results

### Behavioural and neural investigations of the positive reactivity

**Fig. 3:**
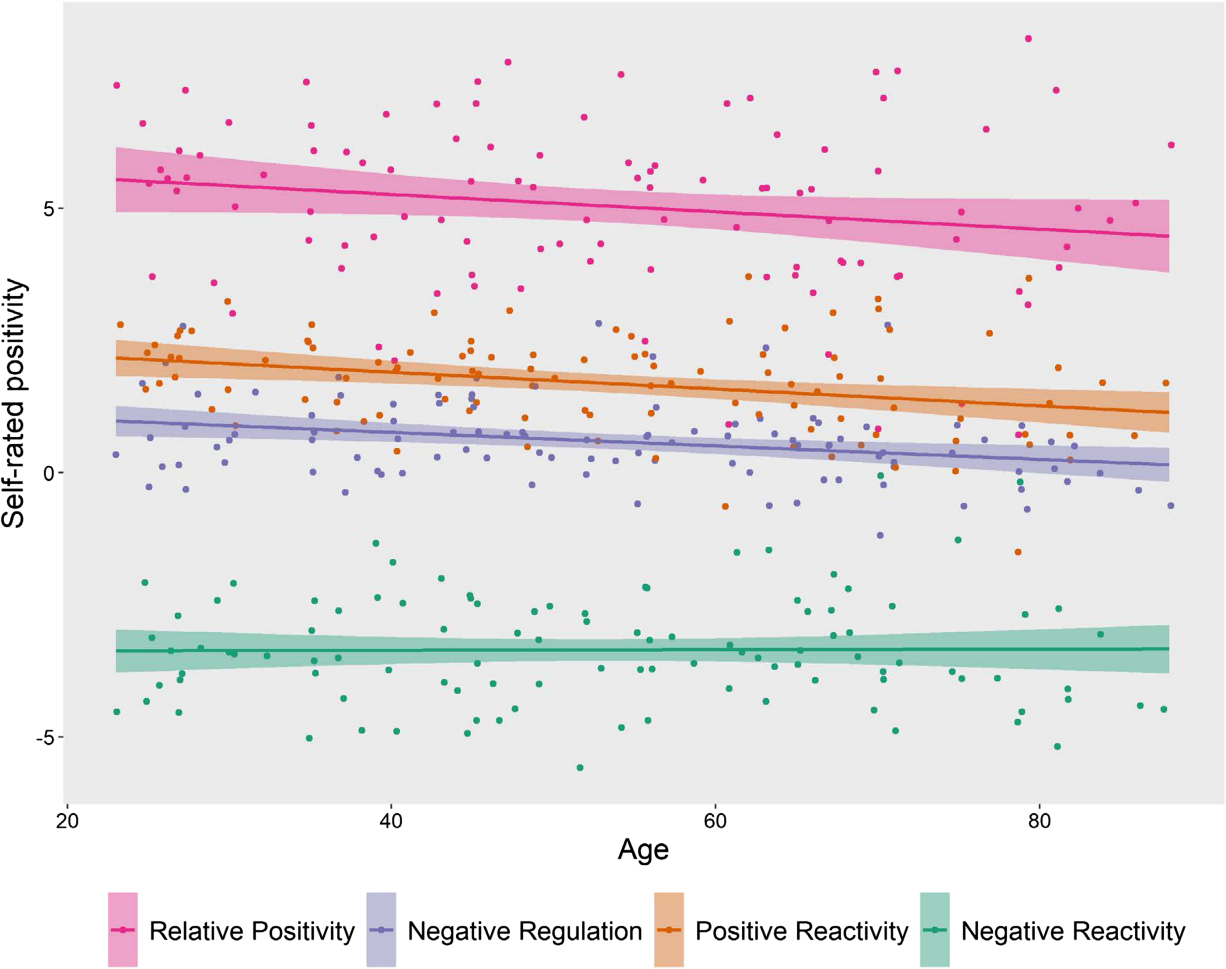
The figure depicts the significant association between age and emotion ratings across Positive Reactivity, Negative Reactivity and Negative Regulation in a multivariate regression analysis, *F* (3, 100) = 6.60, *P* < 0.001, *R^2^* = 0.17. Both Positive Reactivity (orange) and Negative Regulation (blue) show a significant decline with increasing age. Negative Reactivity (green) appears to remain stable across the lifespan (see text for inferential statistics). The figure further shows the non-significant age-related decline in Relative Positivity (pink), which indexes the difference between positive and negative reactivity. Age-related decreases in fluid intelligence did not account for the association between age and positivity or regulatory success (see Supplementary Materials).

The relationships between age and Positive and Negative Reactivity are plotted in Figure 3. In contrast to the prediction from SST, we found no support for increased Positive Reactivity with age in our population-derived sample. In fact, the data revealed a significant age-related *decline* in Positive Reactivity, *β* = −0.016, 95% CI [−0.026; −0.006], *t* = −3.20, *P* = 0.002, *R^2^* = 0.09. Similarly, there was no support for an age-related decrease in Negative Reactivity, *t* < 1, with the data showing a flat profile with age. Relative positivity, measured as self-reported positivity in response to watch positive compared to negative films also declined with age, though the decline was not statistically significant, *β* = −0.016, 95% CI [−0.034; 0.001], *t* = −1.85, *P* = 0.068, *R^2^* = 0.03. The significant age-related decrease in Positive Reactivity was not moderated by gender, level of education or levels of depressive and anxiety symptoms (see Supplementary Materials).

For the fMRI data we utilised a regions of interest (ROI) approach to examine age-related changes in activation in brain regions associated with emotional reactivity and regulation (Buhle *et al.,* 2014). We also conducted exploratory analyses of ROIs associated with reward processing (see Methods)^1^. We found no support for either hypothesised age-related increases in PFC activation or for our exploratory analyses in reward processing regions in the Positive Reactivity or Negative Reactivity conditions. There were also no age-related decreases in amygdalergic activity in the Negative Reactivity condition. Indeed, the only significant effect was a linear, age-related *decrease* in activation in the left middle frontal gyrus—a region of PFC implicated in emotion regulation—in the Positive Reactivity condition, *β* = −0.004, *t* = −2.20, *P* = 0.028, *R^2^* = 0.05. There was no significant association between activity in the middle frontal gyrus activation and positive reactivity scores with (*r* (104) = 0.06, *P* = 0.53) and without (*r* (104) = 0.12, *P* = 0.21) controlling for age.

Previous fMRI studies have found support for the age-related positivity in the form of enhanced activation in emotion-regulation-related brain networks when contrasting the processing of positive material with the processing of negative material (Mather, 2016). To investigate this, we examined activation associated with the watch positive>watch negative contrast. Again, we found no support for an effect of age. In fact, we once more found the opposite pattern of results, with activation in both the left (*β* = −0.005, *t* = −2.02, *P* = 0.046, *R^2^* = 0.04) and right middle frontal gyri (*β* = −0.006, *t* = −2.28, *P* = 0.025, *R^2^* = 0.05), showing linear declines with age. Follow-up whole brain analyses revealed no additional significant effects of age in association with either Positive or Negative Reactivity.

### Behavioural and neural analyses of age-related emotional reappraisal

The relationship between age and Negative Regulation is shown in Figure 3. The data support the prediction of a decreasing capacity with older age to be able to effortfully down-regulate negative affect using reappraisal, *β* = −0.013, 95% CI [−0.021; −0.004], *t* = −3.07, *P* = 0.003, *R^2^* = 0.08. We found no evidence for any age-related changes in activation either across our ROIs or in whole-brain analyses, for the Regulation Negative contrast.

Finally, as the existent literature often compares the effect of age across age groups, we split our sample into three even groups: young (*N* = 35, age range: 23–44 years, *N* = 35, *M* = 33.5 years); middle-aged (*N* = 35, age range: 45–63 years, *M* = 53.1 years) and older (*N* = 34, age range: 64–88 years, *M* = 73.3 years). These exploratory analyses showed no significant age group by condition interaction in a whole-brain comparison.

## Discussion

The age-related positivity effect is one of the most widely replicated findings in the cognitive neuroscience of healthy ageing (Reed *et al.,* 2014), and this age-related improvement in affective functioning stands in stark contrast to research across almost all other domains of cognition where steady declines in functioning are the norm (Hedden and Gabrieli, 2004). The current study examined age-related emotional reactivity and emotion regulation in a large population-derived cohort and found no support for the increased positive emotional reactivity. In fact, emotional positivity in response to positive film clips as well as the ability to effortfully down-regulate negative emotions in response to negative footage, significantly decreased from age 23 to 88 years. At the neural level there was also no support for increased PFC activation when watching positive or negative films or when down-regulating emotions in response to negative films. Instead, decreased positivity across the lifespan was associated with decreased activation in the middle frontal gyrus bilaterally—a region commonly implicated in emotion regulation (Lindquist *et al.,* 2012; Buhle *et al.,* 2014)—suggesting decreased, not increased, engagement of the brain’s control regions with age. Similarly, the failure to find an age-related increase in PFC activation when effortfully down-regulating negative emotions in the current sample is in line with other studies of age-related changes in activation in the neural substrates of emotion regulation (Winecoff *et al.,* 2011; Opitz *et al.,* 2012; Allard and Kensinger, 2014). Using a group (old *vs* young) design these prior studies all showed greater PFC activation in younger adults.

The distinct feature of the present study was the use of population-derived cohort from across the lifespan (Shafto *et al.,* 2014). The current findings are consistent with the hypothesis that the widely replicated positivity effect (Carstensen and DeLiema, 2018), most usually observed when comparing older to younger age groups, at least as it pertains to the study of emotional reactivity and regulation, may be a function of differential reasons or motivations for research participation. For example, (financial) resource acquisition or participation for student course credits in the younger age groups, compared to prosocial engagement with research and its associated positive affective experience (Aknin *et al.,* 2018) in the older adults. These putative differential reasons for participating in ageing studies are in line with SST’s proposed motivational shift from younger to older adulthood.

In line with this motivational account, we propose that once such motivational biases are mitigated by the use of a population-derived study sample, as in the present research, we see a decline in both positivity and effortful emotion regulation with age, congruent with the broader ageing literature (Hedden and Gabrieli, 2004; Craik and Bialystok, 2006). Though these differences could not be fully mitigated in the current study, which required participants to agree to two or more separate testing sessions. Moreover, it should be noted that age accounted for only 5–8% in the observed variance in reported positivity, which is smaller than declines observed in other domains such as for example memory (Henson *et al.,* 2016).

Similarly, despite widespread empirical support for SST, in line with our findings, some other indicators of later-life emotional well-being such as depressive symptoms paint a more mixed picture of affective experiences in older age (Beekman *et al.,* 1995; Jorm, 2000). Population-derived studies have shown an increase in depressive symptoms from middle to old age (Sutin *et al.,* 2013; Tomitaka *et al.,* 2016) or overall invariance across the lifespan but specific older age-related individual variance in depressive symptomatology (Kwon *et al.,* 2017; Schaakxs *et al.,* 2017). Interesting avenues for future research would include prospective studies to investigate whether variation in emotion regulation capacity and its neural substrates across the lifespan are predictive of depressive symptoms and other indicators of emotional well-being in later life, using large population-derived or representative samples.

So far we have interpreted our findings in the context of the SST; however, it should be briefly noted that our results also do not support one of the prominent alternative accounts of age-related increases in positive emotionality: the Aging Brain Model (Cacioppo *et al.,* 2011). The model proposes that the increased positivity found in older age is driven by decreasing amygdalergic reactivity to negative, but not positive, information with age, this was not found in the current sample.

Finally, the following alternative interpretation and limitation should be considered when interpreting the current findings. Namely, an alternative account for the decline in positivity observed in the current study is that the type of positive film clips presented may have been more amusing to the younger individuals. And a limitation of the study was that emotion regulation capacity was only investigated in relation to negative films, and the study therefore cannot speak to potential age-differences in the capacity to up- or down-regulating positive emotions across the lifespan.

In sum, the current study used a population-derived sample from across the life span to investigate positive emotional reactivity embedded in the positivity effect, and its neural correlates, as well as behavioural and neural indices of explicit emotion regulation, using an ecologically-valid film based paradigm. We found no support for increased positive emotional reactivity in ageing, and in fact showed that individuals report feeling less positive in response to positive film clips as a function of increasing age. The decreased positivity was associated with decreased activation in the middle frontal gyrus. Additionally, the ability to effortfully down-regulate negative affect declined with age. Future work in the field should extend and replicate these findings in other population-derived samples.

## Author contributions

S.S., A.J.C. and T.D. were responsible for the conceptualization. S.S., J.S., J.v.B., D.P. and T.D. worked on the methodology. Cam-CAN was responsible for the investigation. S.S. was responsible for the writing of the original draft. S.S., J.S. and T.D. also helped with the writing, review and editing. T.D. and Cam-CAN helped with the funding acquisition. T.D. also contributed with the supervision.

## Conflict of Interests

None declared.

## Supplementary Material

scan-18-391-File003_nsz036

## Endnotes

1 These analyses were suggested during the peer review process and exploratory in nature. We therefore did not correct our significance threshold for these additional exploratory comparisons.
