# Supplementary material for: Age-related decline in positive emotional reactivity and emotion regulation in a population-derived cohort
Source: Soc Cogn Affect Neurosci. 2019 May 23;14(6):623–31. doi: 10.1093/scan/nsz036 (PMC6688446; doi:10.1093/scan/nsz036)
Supplement: scan-18-391-File003_nsz036 [file scan-18-391-file003_nsz036.pdf]

Supplementary Materials

Age-related decline in positive emotional reactivity and emotion regulation in a population-derived cohort

Susanne Schweizer

Jason Stretton

Janna Van Belle

Darren Price

Andrew J. Calder

Cam-CAN

Tim Dalgleish

Supplementary Methods

Emotion Reactivity and Regulation Task

Emotional film clips. The task included ten 30 second film clips in each condition: positive watch, neutral watch, negative watch and negative regulate. The thirty emotional clips were followed by a 5 second washout clip, with the exception of the final clip for each participant which was a positive film clip.

The content of the *Negative* film clips included documentaries about the terrorist attacks of 9/11 (n = 4), a case of child abuse (n = 1), the Gaza conflict including child fatalities (n = 2), Rwanda including fatalities (n = 8); victims of gun violence (n = 1), loss through AIDS (n = 1), child soldiers (n = 1); fictional footage (n = 2) from the American History X curb scene and a dying soldier in Saving Private Ryan. The *Neutral* clips included were documentaries (n = 3) about Alexander the Great, Heidegger, and news editing; a cooking demonstration (n = 1); news reports (n = 3) about a new airport terminal, energy strategies, and market shares; a weather report (n = 1); fictional footage (n = 2) of a cocktail party scene and a scene of younger and older man chatting in a restaurant. The *Positive* clips included compiled footage of funny sporting incidents (n = 2), baby/toddler videos (n = 4), funny animal videos (n = 2), German comedian singing “Men in Black” with Will Smith (n = 1); fictional footage from “10 things I hate about you” (n = 1).

Our normative ratings collected on a different sample (for details see Schweizer et al.(Schweizer, Grahn, Hampshire, Mobbs, & Dalgleish, 2013)) for these films showed that valence ratings differed significantly from each other with a main effect of condition, F (1, 37) = 348.44, $p \leq .001$, d = 8.71. Univariate comparisons showed that negative films had the lowest positivity rating M = 2.33, SD = 0.66, positivity ratings were intermediate

for neutral films *M* = 5.04, *SD* = 0.09, and highest for positive films, *M* = 7.46, *SD* = 0.39. All ratings differed significantly from each other, all *p's* ≤ .001.

Emotional ratings of film clips across individual conditions. There was a significant effect of individual experimental condition on emotion ratings, F (3, 309) = 798.61, $p < .001$, η_p^2=0.88). The films in the positive watch condition were rated as the most positive (*M* = 7.94, *SD* = 0.85) followed by ratings in the neutral watch (*M* = 6.24, *SD* = 0.38), negative regulate (*M* = 3.49, *SD* = 1.04) and negative watch conditions (*M* = 2.89, *SD* = 0.99).

Table S1. Blood-oxygenated level-dependent (BOLD) activation during emotional reactivity and regulation

Contrast	L/R	Label	MNI (X,Y,Z)	*k*	*Z*	*p*
Positive Reactivity						
Positive Watch > Neutral Watch						
	R	Inf. Occipital Gyrus	39 -81 3	8854	Infinite	FWE < 0.05
	R	Precentral Gyrus	45 6 27	2151	Infinite	FWE < 0.05
	L	Mid. Frontal Gyrus	-48 54 9	547	7.66	FWE < 0.05
	L	Precentral Gyrus	-45 3 27	583	7.14	FWE < 0.05
	L	Ant. Insula	-33 24 3	479	6.68	FWE < 0.05
	R	Mid. Frontal Gyrus	36 63 18	355	6.57	FWE < 0.05
	R	Cerebellum	33 -39 -36	13	5.08	FWE < 0.05
Negative Reactivity						
Negative Watch > Neutral Watch						
	R	Sup. Occipital Gyrus	30 -75 24	3011	Infinite	FWE < 0.05

R	Precentral Gyrus	45 6 27	39	5.57	FWE < 0.05
R	Med. Frontal Cortex	9 57 -12	12	5.54	FWE < 0.05
R	Sup. Frontal Gyrus	15 69 24	41	5.40	FWE < 0.05
R	Precuneus	9 -39 45	61	5.34	FWE < 0.05
L	Frontal Pole	-24 66 0	31	5.18	FWE < 0.05
L	Cerebellum	-24 -33 -39	15	5.10	FWE < 0.05
R	Mid. Frontal gyrus	30 36 51	77	5.09	FWE < 0.05
R	Sup. Frontal Gyrus	9 48 54	19	5.03	FWE < 0.05
L	Caudate	-9 9 15	26	4.86	FWE < 0.05
Negative Regulation					
Negative Regulate > Negative Watch					
L	Mid. Frontal Gyrus	-36 54 0	24	3.43	.001 uncorr
Negative Watch > Negative Regulate					
L	Med. Frontal Cortex	-6 33 -18	110	4.31	.001 uncorr
L	Precuneus	-15 -54 15	49	4.02	.001 uncorr
R	Precuneus	21 -54 21	225	3.95	.001 uncorr
L	Inf. Temporal Gyrus	-42 -57 -3	28	3.95	.001 uncorr
L	Supplementary Motor	-12 -6 48	35	3.89	.001 uncorr
L	Sup. Temporal Gyrus	-54 -6 -9	51	3.87	.001 uncorr
L	Sup. Parietal Cortex	-30 -36 42	132	3.76	.001 uncorr
L	Temporal Pole	-63 15 -24	37	3.63	.001 uncorr
R	Temporal Pole	27 15 -27	61	3.55	.001 uncorr
L	Thalamus	-18 -30 0	25	3.45	.001 uncorr
Positive Watch > Negative Watch					
R	Inf. Occipital Gyrus	42 -69 3	1741	Infinite	FWE < 0.05

L	Inf. Occipital Gyrus	-45 -72 6	798	Infinite	FWE < 0.05
L	Supramarginal Gyrus	-57 -39 33	527	6.29	FWE < 0.05
L	Mid. Frontal Gyrus	-39 54 9	328	6.19	FWE < 0.05
R	Precentral Gyrus	45 3 42	139	5.98	FWE < 0.05
L	Mid. Frontal gyrus	-27 -6 48	86	5.89	FWE < 0.05
R	Mid. Cingulate	6 -18 33	122	5.89	FWE < 0.05
R	Supplementary Motor	9 9 60	54	5.78	FWE < 0.05
R	Mid. Frontal Gyrus	54 51 0	79	5.67	FWE < 0.05
L	Anterior Insula	-39 18 6	187	5.33	FWE < 0.05
L	Precuneus	-9 -69 39	29	5.19	FWE < 0.05
R	Frontal Operculum	51 15 0	135	5.07	FWE < 0.05
Negative Watch > Positive Watch					
L	Mid. Temporal Gyrus	-57 -6 -15	395	Infinite	FWE < 0.05
R	Calcarine Cortex	12 -90 3	59	7.04	FWE < 0.05
R	Mid. Temporal Gyrus	60 -6 -15	84	6.02	FWE < 0.05
L	Calcarine Cortex	-9 -84 6	87	5.86	FWE < 0.05
L	Med. Frontal Gyrus	-3 48 -15	182	5.73	FWE < 0.05
L	Precuneus	-9 -63 15	28	5.44	FWE < 0.05

Table S1. FWE < 0.05 = family-wise error corrected at $p < .05$; .001 uncorr = significance threshold uncorrected for multiple comparisons at $p < .001$. Ant. = anterior; inf. = inferior; med. = medial; mid. = middle; sup. = superior.

Structural analyses

As described in Taylor et al., the T1 image was initially coregistered to the MNI template, and the T2 image was then coregistered to the T1 image using a rigid-body (6-

df) linear transformation. The coregistered T1 and T2 images were used in a multi-channel segmentation (SPM12 Segment, based on "New Segment" in SPM8[1]) routine in order to extract probabilistic maps of 6 tissue classes: Gray Matter (GM), White Matter (WM), cerebrospinal fluid (CSF), bone, soft tissue, and residual noise. The native-space GM and WM images for all participants who passed quality-control checks were then submitted to diffeomorphic registration (DARTEL[2]) to create group template images. The group template was then normalised to the MNI template via an affine transformation, and combined normalisation parameters (native to group template and group template to MNI template) were applied to each individual participant's GM and WM images. From this stage, the structural analysis stream followed two branches: For voxel-based morphometry analysis, individual normalised GM and WM images were smoothed (8 mm FWHM Gaussian kernel)

The GM images (N = 104) were then entered into a second level one-sample t-test. We then used the MarsBar ROI tool box to extract the GM volumes from the six functional ROIs applied to the fMRI data and entered the values of each region into a multiple linear regression with age as the independent variable.

Supplementary Results

Structural results

All six of the ROIs showed declining GM with increasing age; Left MFG (β = –0.408, t = –4.51, p = .000, R^2 = .41); Right MFG (β = –0.335, t = –3.58, p = .000, R^2 = .33); Left amygdala (β = –0.464, t = –5.29, p = .000, R^2 = .46); Right amygdala (β = –0.371, t = –4.04, p = .000, R^2 = .14); Right Medial FG (β = –0.509, t = –5.97, p = .000, R^2 = .51); Right IFG (β = –0.421, t = –4.68, p = .000, R^2 = .42).

The positivity effect is not moderated by gender, education or depression

The age related decline in positivity was not moderated by gender, β = 0.01, *t* = 0.64, *p* = .53, level of education, β = 0.00, *t* = 0.28, *p* = .78 or symptoms of depressed or anxious mood, β = 0.00, *t* = 0.35, *p* = .73.

The effect of fluid intelligence on positivity and emotion regulation.

Fluid intelligence as measured by a matrix reasoning task (Cattell, 1971), did not significantly predict participants' Positive Reactivity, β = –0.02, *t* = –1.09, *p* = .28, R^2 = .01; Negative Reactivity β = –0.01, *t* = –0.60, *p* = .55, R^2 = .00 or regulation capacity β = 0.01, *t* = –0.58, *p* = .55, R^2 = .00.
